# Time-to-completed-imaging, survival and function in patients with spinal epidural abscess: Description of a series of 34 patients, 2015–2018

**DOI:** 10.1186/s12913-020-4973-5

**Published:** 2020-02-14

**Authors:** Caroline King, Cameron Fisher, Patrick C. M. Brown, Kelsey C. Priest, Mary Tanski, Peter Sullivan

**Affiliations:** 1 School of Medicine, Department of Biomedical Engineering, Oregon Health & Science University, Portland, OR USA; 2 School of Medicine, Oregon Health & Science University, Portland, OR USA; 3 School of Public Health, Oregon Health & Science University-Portland State University, Portland, OR USA; 4 Department of Emergency Medicine, Oregon Health & Science University, Portland, OR USA; 5 Department of Internal Medicine, Oregon Health & Science University, Portland, OR USA

**Keywords:** Spinal epidural abscess, Intravenous drug use, Substance use disorder, Homeless

## Abstract

**Introduction:**

Spinal epidural abscess (SEA) is a rare and life-threatening infection with increasing incidence over the past two decades. Delays in diagnosis can cause significant morbidity and mortality among patients.

**Objective:**

The objective of this study was to describe trends in time-to-imaging and intervention, risk factors, and outcomes among patients presenting to the emergency department with SEA at a single academic medical center in Portland, Oregon.

**Methods:**

This retrospective cohort study analyzed data from patients with new SEA diagnosis at a single hospital from October 1, 2015 to April 1, 2018. We describe averages to time-to-imaging and interventions, and frequencies of risk factors and outcomes among patients presenting to the emergency department with SEA.

**Results:**

Of the 34 patients included, 7 (20%) died or were discharged with plegia during the study period. Those who died or were discharged with plegia (*n* = 7) had shorter mean time-to-imaging order (20.8 h versus 29.2 h). Patients with a history of intravenous drug use had a longer mean time-to-imaging order (30.2 h versus 23.7 h) as compared to those without intravenous drug use. Patients who died or acquired plegia had longer times from imaging completed to final imaging read (20.9 h versus 7.1 h), but shorter times from final imaging read to surgical intervention among patients who received surgery (4.9 h versus 46.2 h). Further, only three (42.9%) of the seven patients who died or acquired plegia presented with the three-symptom classic triad of fever, neurologic symptoms, and neck or back pain.

**Conclusions:**

SEA is a potentially deadly infection that requires prompt identification and treatment. This research provides baseline data for potential quality improvement work at the study site. Future research should evaluate multi-center approaches for identifying and intervening to treat SEA, particularly among patients with intravenous drug use.

## Introduction

### Background/rationale

Spinal epidural abscess (SEA) is a rare but life-threatening infection; recent epidemiologic studies report an increased incidence of SEA over the past two decades [1, 2]. Explanations for this increase are multifactorial and may include increased use of invasive spinal procedures, improved sensitivity and availability of diagnostic tools (e.g., magnetic resonance imaging), population age, and the opioid-related overdose epidemic, specifically, an increase in intravenous drug use (IVDU), which is a SEA risk factor [1, 2] . The commonly taught clinical triad for SEA is fever, neurologic status changes, and back or neck pain; however, previous research demonstrates that patients rarely present with all three symptoms [3, 4] and 20% of patients with SEA have no associated risk factors [1]. Thus, risk-factor-based screening remains of limited clinical utility. Unsurprisingly, SEA is frequently misdiagnosed or under-diagnosed [3, 4], and when successfully diagnosed, a delay to diagnosis is common.

These delays in SEA diagnosis may cause significant morbidity and mortality among patients [3, 4]. Neurological outcomes depend on the symptom severity at presentation and duration prior to diagnosis [4]. Mortality is also related to delayed recognition emphasizing the importance for timely diagnosis [1, 3]. Little research on interventions to reduce delays has been conducted to date. This study sought to evaluate a cohort of patients diagnosed with SEA at a single academic health center in Portland, Oregon. Here, we describe trends in time-to-imaging and intervention, risk factors, and outcomes among people presenting to the emergency department (ED) with SEA.

## Methods

### Study design and setting

This retrospective cohort analysis was completed at the Oregon Health & Science University (OHSU) hospital in Portland, Oregon. OHSU is a 550-bed teaching hospital, Level-1 trauma Center, that is Oregon’s sole academic health center. This case series includes OHSU patient encounters between October 1, 2015 and April 1, 2018.

### Participants

Patients were at least 18 years old at the time of ED presentation and were diagnosed with a SEA during the encounter (ICD-10 code G06.1). Patients who were transferred to OHSU after receiving imaging at an outside hospital were excluded, as were patients who were admitted directly to an inpatient floor at OHSU. This allowed us to better capture how the emergency department at our institution evaluated patients with suspected SEA.

### Variables

The primary variable of interest was time in hours from diagnostic imaging order to the time that imaging was completed. For patients with multiple visits to the hospital prior to imaging, the earliest visit hour without a 72-h gap until or between subsequent visits was considered ED presentation time. Additional time intervals collected and reported include: 1) time from ED presentation to imaging order; 2) time from imaging order to imaging complete; 3) time from imaging completion to imaging final with radiology reading; and 4) time from final imaging read to surgical intervention. The time at which the spinal abscess pathology cultures were sent was a proxy for surgical intervention. We also used the imaging order time that confirmed the spinal epidural abscess for evaluation (versus, for example, a CT that did not identify a SEA before an MRI was completed).

At OHSU, treating physicians worked with surgical and infectious disease specialists to make decisions regarding surgical versus medical intervention in treating SEA. To report how often surgical versus medical interventions were pursued, we also identified the proportion of patients who were treated surgically versus with antibiotics. For patients with suspected infection, the OHSU emergency department protocol was to treat septic patients with broad-spectrum antibiotics; patients who were clinically stable may wait for surgical biopsy or culture results before receiving antibiotic treatment.

### Demographics

Collected demographics and covariates are reported in Table 1. Variables reported were identified via chart abstraction or from administrative billing data. Data from administrative data included age (years), sex (male/female), race (Black/Caucasian/Asian/American Indian or Alaska Native/Other), insurance status (any Medicaid/any Medicare/private insurance/no insurance), history of alcohol use disorder (ICD-10 code F10.x; y/n), history of HIV/AIDS (ICD-10 codes B20, Z21; y/n), history of Diabetes Mellitus (ICD-10 codes E11.x; y/n), history of Chronic Kidney Disease (ICD-10 codes I12.x, I13.x, N18.x; y/n), currently on dialysis (ICD-10 code Z99.2; y/n), active malignancy (ICD-10 codes C00-C96; y/n), history of homelessness (ICD-10 code Z59.0; y/n), seen at OHSU or elsewhere in previous 72 h (y/n), left against medical advice within previous 72 h (y/n), and culture results of abscess (staph aureus/methicillin resistant staph aureus/*Propionibacterium acnes*/strep pyogenes/aggregatibacter aphrophilus/no growth/no culture).

**Table 1:** Demographics of patients diagnosed with spinal epidural abscess in a retrospective cohort study at Oregon Health & Science University, 2015–2018

	Plegia or death (*n* = 7)	No plegia or death (*n* = 27)
Age	55.1 (16.2)	49.2 (15.5)
Sex		
Female	3 (42.9)	10 (37.0)
Race		
White	5 (71.4)	23(85.2)
African-American	0	2 (7.4)
Asian	1 (14.3)	0
American Indian/Alaska Native	0	0
Other/declined	1 (14.3)	2 (7.4)
Insured		
Any Medicare	5 (71.4)	24 (88.9)
Any Medicaid	3 (42.9)	8 (29.6)
Private Insurance	1 (14.3)	2 (7.4)
No Insurance	0	1 (3.7)
Military Insurance	0	7 (25.9)
Fever in first 24 h	4 (57.1)	17 (63.0)
Back or neck pain in first 24 h	7	23 (85.2)
Paresthesia, focal weakness or abnormal neuro exam in first 24 h	6 (85.7)	18 (66.7)
Intravenous drug use	2 (28.6)	18 (66.7)
Alcohol use disorder	2 (28.6)	4 (14.8)
Seen by healthcare facility in 72 h prior to presentation	2 (28.6)	7 (25.9)
Left against medical advice in previous 72 h	1 (14.3)	1 (3.7)
HIV/AIDS	0	1 (3.7)
Homeless	1 (14.3)	7 (25.9)
Diabetes	3	6
On dialysis	0	1
Chronic Kidney Disease	1	2
Active malignancy	0	1

*values shown are n(%) or mean (SD)

Data identified and abstracted from chart review include presence of a fever (temperature > 99.5) within first 24 h of hospital contact (y/n), presence of paresthesia, focal weakness or abnormal neurologic exam within first 24 h of hospital contact (y/n), presence of neck or back pain in first 24 h of hospital contact (y/n), history of injection drug use (y/n), and interval-based times to imaging and surgical intervention. The number of patients presenting with combinations of fever, back or neck pain, and abnormal neurologic exam in the first 24 h are also reported; abnormal neurologic exam included any abnormality listed in the neurologic and motor exams in documented review of systems, and any listed neurologic or motor deficit in the clinical summary. Demographic variables were determined in consultation with treating teams and through a literature review, which identified risk factors for mortality from SEA, as well as for risk of recurrence [5, 6]. Finally, we also report the type of imaging that first detected a spinal epidural abscess (type of MRI versus CT).

### Outcome

The primary outcome was a composite variable of plegia at discharge or in-hospital death. Plegia was identified using ICD-10 codes G81, G82, G83.1, G83.2, and G83.3 and verified via chart review by a reviewer blinded to ICD-10 code information. This composite variable was agreed upon by study team as a relevant study outcome with implications for care delivery for SEA patients. For patients who left the hospital and subsequently returned within 72-h, the outcome was recorded from the last encounter with less than a 72-h gap between encounters.

### Analysis

Because SEA is rare, we anticipated a low number of cases at our institution meeting our inclusion criteria. We describe our cohort of patients using measures of central tendency (means and medians) and frequencies of variables collected, characterized by the dichotomous outcome variable (plegia or death at discharge).

### Consent and ethical considerations

This project was approved by OHSU’s Institutional Review Board (IRB #00018422).

## Results

**Fig. 1:**
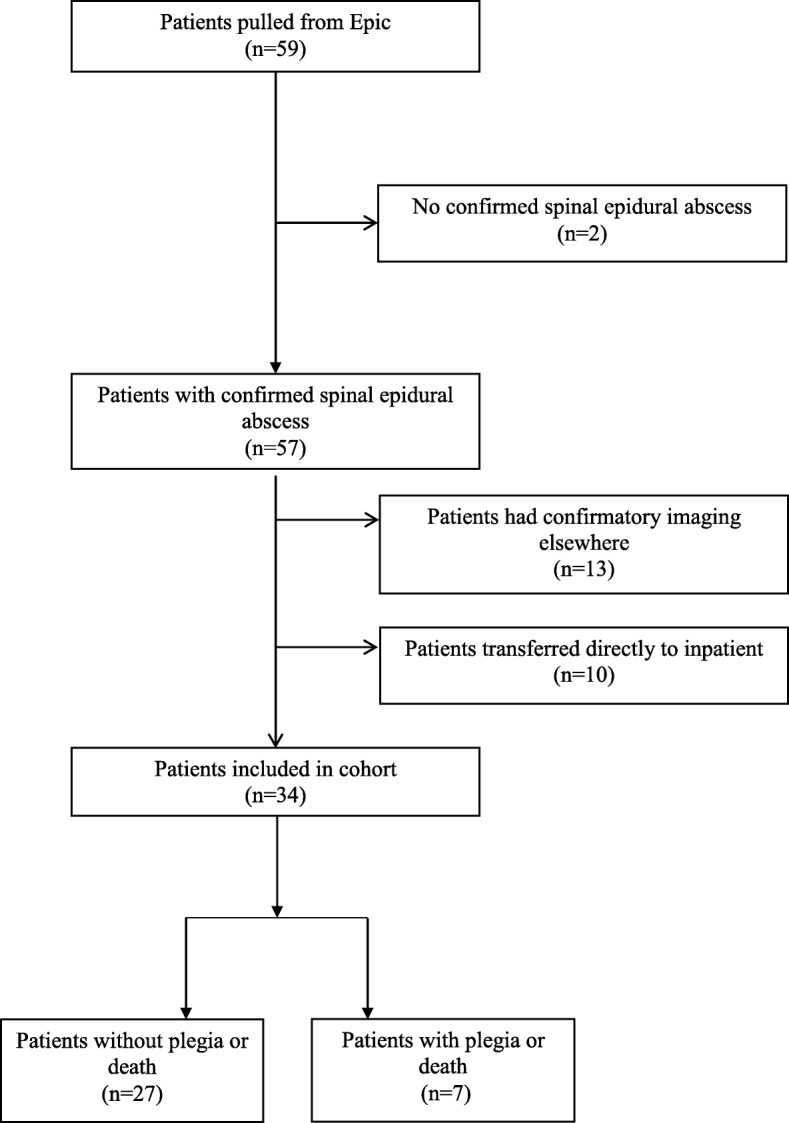
Participant enrollment flowchart

Of the 34 patients included in the cohort, three died and four were discharged with plegia (Fig. 1). Of those seven, two had a history of IVDU (Table 1). All seven presented initially with back or neck pain within the first 24-h of ED contact; six (85.7%) had an abnormal neurologic exam, and four (57.1%) had a fever greater than 99.5 degrees Fahrenheit (Table 2). Three (42.9%) of the seven patients who died or acquired plegia presented with the classic triad of fever, back or neck pain, and neurologic changes. As described above, we included patients with surgical and non-surgical treatment plans for SEA; of the 34 patients, 24 patients underwent surgical intervention for SEA (70.6%).

**Table 2:** Table of patients presenting with combinations of fever, back or neck pain, or abnormal neurologic exam in the first 24 h of care for spinal epidural abscess, 2015–2018

	Plegia or death (*n* = 7)	No plegia or death (*n* = 27)
Fever only	4 (57.1)	17 (63.0)
Back or neck pain only	7	23 (85.2)
Abnormal neuro exam only	6 (85.7)	18 (66.7)
Fever and back/neck pain	4 (57.1)	16 (59.3)
Fever and abnormal neuro exam	3 (42.9)	11 (40.7)
Back/neck pain and abnormal neuro exam	6 (85.7)	14 (51.9)
Fever, back/neck pain and abnormal neuro exam	3 (42.9)	10 (37.0)

Patients who died or acquired plegia had a mean time-to-imaging order and time from imaging order to completed imaging shorter than those who survived without plegia (20.8 h versus 29.2 h, and 11.5 h to 14.2 h, respectively) (Table 3). Among people with a history of IVDU, the mean time from ED presentation to imaging order was longer (30.2 h versus 23.7 h) than those without a history of IVDU. Patients who died or acquired plegia had longer times from imaging completed to final imaging read (20.9 h versus 7.1 h), but shorter times from final imaging read to surgical intervention among patients who received surgery (4.9 h versus 46.2 h).

**Table 3:** Interval imaging times in hours for patients with spinal epidural abscess in a retrospective cohort study at Oregon Health & Science University, 2015–2018

Time from:	Plegia or death (*n* = 7)	No plegia or death (*n* = 27)
ED presentation to imaging order	20.8 (37.0)	29.2 (44.9)
Imaging order to imaging completed	11.5 (8.6)	14.2 (23.3)
Imaging completed to final imaging read	20.9 (33.6)	7.1 (7.3)
Final imaging read to surgical intervention (*n* = 5 for plegia/died, *n* = 19 for survived)	4.9 (9.4)	46.2 (52.1)

All patients had diagnostic imaging completed that identified their spinal epidural abscess (Table 4). The most commonly ordered diagnostic images were MRI of the lumbar spine with and without contrast (*n* = 7, 20.6%) and MRI of the total spine with and without contrast (n = 7, 20.6%). Four patients of 34 had their SEA identified by CT (11.8%).

**Table 4:** Type of imaging first detecting spinal epidural abscess among patients at Oregon Health & Science University, 2015–2018

Type of imaging	Number of patients *n* = 34 (%)
CT abdomen and pelvis with contrast	1 (2.9%)
CT neck soft tissue with contrast	2 (5.9%)
CT spine total w contrast	1 (2.9%)
MRI spine cervical with and without contrast	2 (5.9%)
MRI spine lumbar without contrast	2 (5.9%)
MRI spine lumbar with and without contrast	7 (20.6%)
MRI spine thoracic lumbar with and without contrast	1 (2.9%)
MRI spine thoracic without contrast	3 (8.8%)
MRI spine thoracic with and without contrast	2 (5.9%)
MRI spine total with and without contrast	3 (8.8%)
MRI total spine without contrast	3 (8.8%)
MRI total spine with and without contrast	7 (20.6%)

Of the 13 patients with positive cultures who used intravenous drugs and received surgical intervention, 11 patient samples grew either staph aureus or methicillin-resistant staph aureus, as expected (Table 5). The remaining two patients’ cultures grew propionibactirum acnes and aggregatibacter aphrophilus. Among the seven patients with positive cultures without IVDU history, five grew staph aureus, one grew methicillin resistant staph aureus, and one grew strep pyogenes.

**Table 5:** Culture results from patients with spinal epidural abscess with and without a history of intravenous drug use in a retrospective cohort study at Oregon Health & Science University, 2015–2018

	History of intravenous drug use (*n* = 20)	No history of intravenous drug use (*n* = 14)
Aggregatibacter aphrophilus	1 (5.0)	0
*Staphylococcus aureus*	4 (20.0)	5 (35.7)
Methicillin-resistant Staphylococcus aureus	7 (35.0)	1 (7.1)
*Propionibacterium acnes*	1 (5.0)	0
*Streptococcus pyogenes*	0	1 (7.1)
No growth	2 (10.0)	1 (7.1)
No culture	6 (30.0)	4 (28.6)

## Discussion

The study objective was to describe averages in time-to-imaging and intervention and frequencies of risk factors and outcomes, among people with SEA. Of the 34 patients in our cohort, 7 (20%) died or were discharged with plegia during the study period. Those who died or were discharged with plegia (*n* = 7) had shorter mean time-to-imaging order; patients with a history of IVDU had a longer mean time-to-imaging order versus those without IVDU.

Estimating 303,243 patient visits per year at OHSU overall [7], in this time period we observed an incidence of 1.12 per 10,000 visits for new SEA (excluding patients with SEA diagnosed elsewhere that were transferred to OHSU)). In our study, four patients were discharged with plegia (11.8%) and three died in the hospital (8.8%). Recent work cites plegia rates to be around 22% following SEA, with mortality between 3 and 25% [8]. Our rates of plegia and mortality among newly diagnosed patients were lower than published averages.

Among patients with SEA who developed plegia or died, times from ED presentation to imaging order to imaging complete, and from final imaging read to surgical intervention, were all shorter than for patients who did not develop plegia or die. However, time from imaging completed to final imaging read were nearly 2.5 times longer for patients who died or developed plegia. While this could suggest this is a meaningful interval to address for patients screened for SEA, perhaps equally important is the overall time from ED presentation to surgical intervention for patients in both groups.

Among patients who died or developed plegia, the average time from presentation to surgical intervention was 58.1 h; for patents without plegia or death, the average time was nearly twice that, at 96.7 h. Recent research has advocated for advancing patients to surgery more quickly when suspicious for a SEA [9]. Better achieving this may mean expediting evaluation and treatment for patients with SEA; times to imaging and intervention should be explored as potential quality measures.

Patients with a history of IVDU had longer wait times than patients without a history of IVDU from the time they presented to the ED to the time of imaging order. This is an important finding that should be evaluated in larger studies. The reasons for delayed care could be related to multifactorial and complex contributions related to discrimination, stigma, and limited education on how to care for patients with substance use disorders (e.g., clinical management of acute intoxication or withdrawal) among ED providers. Some of the barriers to care for persons with IVDU in the ED, particularly as they relate to stigma, are described elsewhere [10–12].

Fewer than half of the patients who died or acquired plegia presented with the classic triad of fever, back or neck pain, and neurologic changes. This was unsurprising; though the classic triad for SEA exists, patients are rarely documented presenting with all three symptoms [3, 4]; one paper identified all three symptoms in only 8% of patients [13]. However, all seven patients who developed plegia or died presented initially with back or neck pain. The prognostic value of back or neck pain alone in supporting further work-up for SEA should be examined in future work.

Most patients had an MRI ordered which diagnosed their SEA; only four patients had CT scans completed that identified their SEA. MRIs are the modality of choice in diagnosing SEA, as they allow for better exploration of soft tissue and may be more sensitive at identifying SEA [14]. Reviews highlight that CT should only be ordered to diagnose SEA where MRI is unavailable or contraindicated [15], but surgical teams may prefer to have both MRI and CT at the time of surgical intervention to better evaluate invasion into bony spaces.

This paper was strengthened by the identification of only new SEA cases within our health care system, thus limiting cases to those truly new to healthcare providers. This work also identifies a new, hard to capture variable for consideration in the evaluation of care quality for patients with SEA: time from imaging to surgical intervention. However, this paper was limited by the by the available patient population because of the criteria for cohort enrollment; specifically, our requirement that patients not receive outside imaging prior to transfer reduced our sample size, though it did allow us to accurately report basic information about time-to-imaging among this cohort. Further, around 40% of our cohort did not have IVDU as a primary risk factor for SEA. While we include some additional co-morbid conditions including active malignancy, HIV, and diabetes, we did not request the extraction of lab data (white cell counts, erythrocyte sedimentation rate, and C-reactive protein) or information about the extent or location of abscesses, which may have implications for time from presentation to diagnosis. Further, we used ICD-10 codes to identify some co-morbid conditions and to cull our initial sample of patients with SEA. It is likely that some patients with conditions do not have appropriate ICD-10 codes in their charts, and we may be missing co-morbid conditions or cases of SEA. Finally, because the study design did not have sufficient power for univariate or multivariate analyses, confounding variables such as patient comorbidities and severity of presenting illness were not directly accounted for in reporting central tendencies; thus, it is possible that the seven patients with mortality and poor morbidity outcomes had shorter mean time-to-imaging because they presented as “sicker” than the other patients.

There are important implications from this work. Future research should evaluate multi-center approaches for identifying and intervening to treat SEA, particularly among patients with IVDU, specifically to understand what delays may exist for patients and how to decrease those delays. Additional analyses should include evaluations of time intervals from patient presentation through intervention, though this information may be challenging to obtain; this study obtained imaging information through chart review versus automated data collection.

SEAs are serious infections causing significant morbidity and mortality among a growing patient population in the United States [1]. While there is consensus that time to identification and intervention likely impact survival and outcomes, limited research exists to evaluate potential places for intervention to improve time to identification and intervention among patients presenting to the ED with SEA. We hope to build on this research by following forward a cohort of patients with SEA at OHSU. Finally, the authors of this paper plan work together with an interdisciplinary team of providers to identify targets from this data to reduce the overall wait times for imaging to identify SEA.

## Data Availability

The datasets used and analysed during the current study available from the corresponding author on reasonable request.

## Abbreviations

ED: Emergency department
IVDU: Intravenous drug use
OHSU: Oregon Health & Science University
SEA: Spinal epidural abscess
